# Matrix metalloproteinase 7 contributes to intestinal barrier dysfunction by degrading tight junction protein Claudin-7

**DOI:** 10.3389/fimmu.2022.1020902

**Published:** 2022-10-04

**Authors:** Ying Xiao, Haifeng Lian, Xiaoying S. Zhong, Srikruthi S. Krishnachaitanya, Yingzi Cong, Roderick H. Dashwood, Tor C. Savidge, Don W. Powell, Xiaowei Liu, Qingjie Li

**Affiliations:** ^1^ Department of Gastroenterology, Xiangya Hospital, Central South University, Changsha, China; ^2^ Division of Gastroenterology, Department of Internal Medicine, University of Texas Medical Branch at Galveston, Galveston, TX, United States; ^3^ Yantai Affiliated Hospital of Binzhou Medical University, Yantai, China; ^4^ Department of Microbiology and Immunology, University of Texas Medical Branch at Galveston, Galveston, TX, United States; ^5^ Center for Epigenetics & Disease Prevention, Texas A&M College of Medicine, Houston, TX, United States; ^6^ Texas Children’s Microbiome Center, Baylor College of Medicine, Houston, TX, United States

**Keywords:** inflammatory bowel disease, ulcerative colitis, tight junctions, permeability, matrilysin

## Abstract

**Background:**

Previous studies implicated matrix metalloproteinases (MMPs), such as MMP-7, in inflammatory bowel diseases (IBD) by showing increased activity during inflammation of the gut. However, the pathophysiological roles of MMP-7 have not been clearly elucidated.

**Methods:**

The expression of MMP-7 was assessed in colonic biopsies of patients with ulcerative colitis (UC), in rodents with experimental colitis, and in cell-based assays with cytokines. Wild-type and MMP-7-null mice treated with dextran sulfate sodium (DSS) or trinitrobenzene sulfonic acid were used for determining the pro-inflammatory function(s) of MMP-7 *in vivo*.

**Results:**

MMP-7 was highly expressed in patients with UC and in rodents with experimental colitis. IL-1β, IL-4, IL-13, TNFα, or lipopolysaccharide enhanced MMP-7 expression in human colonic epithelial cells, rat colonic smooth muscle cells, and THP-1-derived macrophages. Active MMP-7 degraded tight junction protein Claudin-7 in epithelial cells, cleaved recombinant Claudin-7 in cell-free system, and increased Caco-2 monolayer permeability. Immunostaining of colon biopsies revealed up-regulation of MMP-7 and reduction of Claudin-7 in UC patients. Compared to wild-type mice, *Mmp7* ^-/-^ mice had significantly less inflammation in the colon upon DSS insult. DSS-induced alterations in junction proteins were mitigated in *Mmp7* ^-/-^ mice, suggesting that MMP-7 disrupts the intestinal barrier. MMP-7 antibody significantly ameliorated colonic inflammation and Claudin-7 reduction in 2 different rodent models of colitis.

**Summary:**

MMP-7 impairs intestinal epithelial barrier by cleavage of Claudin-7, and thus aggravating inflammation. These studies uncovered Claudin-7 as a novel substrate of MMP-7 in the intestinal epithelium and reinforced MMP-7 as a potential therapeutic target for IBD.

## Graphical Abstract

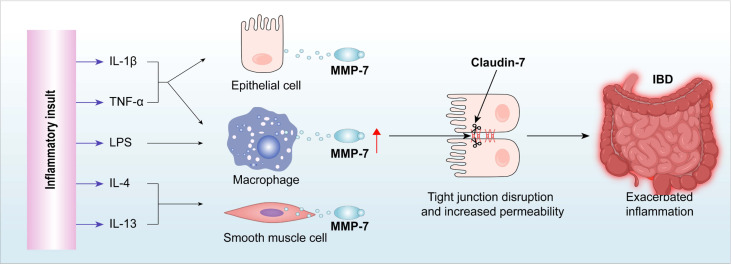

## Introduction

Inflammatory bowel disease (IBD), including Crohn’s disease (CD) and ulcerative colitis (UC), involves interactions between environmental factors and microbiota in the intestinal lumen and inappropriate host immune responses in genetically susceptible individuals (1). At the interface between the mucosal immune system and the external luminal environment, small amounts of antigens are sampled by the epithelium as an important surveillance mechanism (2). On the other hand, excessive uptake of antigens can lead to hyper-activation of the immune system, which can result in mucosal inflammation (3). The ability to control antigen uptake is denoted as a key intestinal barrier function (4). Besides the intestinal epithelial layer, there are other components that constitute the intestinal mucosal barrier: the outer mucus layer, which includes not only secreted mucin and antibacterial substances, but also the commensal microbiota; and the inner subepithelial component, which consists of the innate and adaptive immune system (5). Contacts between adjacent epithelial cells are made of tight junctions (TJ), adherens junctions, and desmosomes and play a crucial role in regulating the intestinal mucosal barrier. Tight junctions appear as a series of appositions or “kissing points” between neighboring cells to form a continuous seal and are the primary determinant of paracellular permeability (6). Adherens junctions are composed primarily from cadherins and provide strong mechanical attachments between adjacent cells. Tight and adherens junction proteins have been implicated in the pathogenesis of IBD (7, 8).

The epithelial TJ is a key structure that regulates paracellular transport of macromolecules. The disruption of the intestinal TJ barrier and the subsequent infiltration of toxic molecules in the lumen induce perturbations of the mucosal immune system and inflammation, which can act as a trigger for the development of intestinal and systemic diseases (9). Although it is unknown whether barrier dysfunction precedes pathology or is caused by active inflammation, the enhanced intestinal TJ disruption observed in patients with IBD suggests that dysregulation of TJ barrier integrity may precipitate or promote IBD progression (10, 11).

There are four identified tight junction proteins, i.e. occludin, claudins, junctional adhesion molecule, and tricellulin; claudins and occludin are the most important TJ proteins that control the vital function of the epithelial cells albeit some functions of the transmembrane tight junction proteins rely on interactions with components of the complex cytosolic plaque underlying the junctional membrane, including zonula occludens 1 (ZO1), ZO2, and ZO3 (12). Claudins are considered as the backbone or pillar of tight junction strand and 27 claudins have been discovered thus far (12–14). Recent studies in knockout mice provided direct evidence that claudins play a key role in barrier formation and paracellular permeability selectivity in various tissues (15–18). Notably, most Claudin-7 knockout mice die within 9 days of birth because of severe intestinal defects that included mucosal ulcerations, epithelial cell sloughing, and inflammation. Electron microscopy showed intercellular gaps and loose cellular matrix in *Cldn7* ^−/−^ mice (19, 20), underscoring the importance of Claudin-7 in the intestinal epithelial barrier.

Matrix metalloproteinases (MMP) are a group of zinc-dependent endopeptidases with a distinct role in extracellular matrix degradation and remodeling. Under physiological conditions, MMPs are generally in the latent form, but are also involved in normal tissue turnover. MMP function is inhibited by tissue inhibitors of metalloproteinases (TIMPs). In the inflamed gut of IBD patients, however, MMPs are produced in excess, beyond effective TIMP blockade, thereby making a major contribution to intestinal mucosal damage (21). Matrix metalloproteinase-7 (MMP-7), also called matrillysin-1, is the smallest MMP with proteolytic activity against a broad range of substrates, including pro-TNFα and Galectin-3 (22). MMP-7 expression was found to be increased in the inflamed colonic mucosa of IBD (23). However, it is not known if MMP-7 cleaves gut epithelial TJ proteins in IBD.

We hypothesized that MMP-7 plays a key role in prolonging/exacerbating inflammation in IBD by disrupting the intestinal epithelial TJ. Our results show that MMP-7 is highly expressed in the colon of patients with UC and murine models of colitis. MMP-7 impairs the intestinal epithelial barrier by cleaving TJ protein Claudin-7. *In vivo* studies with MMP7-null mice provided evidence that MMP-7 mediates epithelial barrier dysfunction, and that MMP-7 deficiency ameliorates colitis. Furthermore, treatment with MMP-7 monoclonal antibody improves intestinal barrier function and mitigates inflammation in dextran sulfate sodium (DSS)-treated mice and trinitrobenzene sulfonic acid (TNBS)-treated rats, showing therapeutic potential. Our findings taken together establish MMP-7 as an important player in IBD pathogenesis and a promising therapeutic target for the treatment of IBD.

## Materials and methods

### Reagents

Recombinant human TNFα (Cat. # Z01001) and IL-1β (Cat. # Z02922) were purchased from GenScript (Piscataway, NJ). Recombinant human IL-2 (Cat. # 10453-IL), IL-4 (Cat. # 204-IL), IL-6 (Cat. # 206-IL), IL-12 (Cat. # 219-IL), IL-13 (Cat. # 213-ILB), IL-17A (Cat. # 1955-IL), IL-22 (Cat. # 782-IL), and IL-23 (Cat. # 1290-IL) were purchased from R&D Systems (Minneapolis, MN). Active human MMP-7 (Cat. # 44-427-0100UG) and lipopolysaccharide (LPS, Cat. # L4391) were obtained from MilliporeSigma (Burlington, MA). Claudin-7 expression plasmid pCMV6-CLDN7 (Cat. # SC121707) was purchased from OriGene Technologies (Rockville, MD).

### Cell culture

Fetal human cells (FHC) (Cat. *#* CRL-1831), CCD841 CoN (Cat. # CRL-1790), and Caco-2 (Cat. # HTB-37) cells purchased from American type culture collection (ATCC, Manassas, VA) were maintained in DMEM supplemented with 10% FBS and 0.1% penicillin-streptomycin solution at 37°C in a humidified atmosphere of 5% CO_2_ (8). THP-1 (Cat. # TIB-202, ATCC) cells were maintained in RPMI 1640 containing 10% FBS and 0.1% penicillin-streptomycin solution and treated with 100 ng/mL phorbol 12-myristate 12-acetate for 3 days to induce monocytic differentiation. Cells at 80-90% confluency were subjected to 24-hour treatment with cytokines.

Freshly dispersed rat colonic smooth muscle cells (RCSMC) were plated at a concentration of 5 × 10^5^ cells/mL, incubated in a CO_2_ incubator at 37°C, and passaged after 7 to 10 days. The primary cells were subjected to treatment after reaching 80 to 90% confluency (24).

Over-expression of MMP-7 in CCD841 CoN cells was done by transient transfection with Lipofectamine 2000 (ThermoFisher Scientific, Waltham, MA) and the cells were harvested for protein isolation and Western blotting 48 hours later. Stable Caco-2 cell line overexpressing human Claudin-7 was generated by transfection of pCMV6-CLDN7 using Lipofectamine 2000, followed by selection with G418.

### Animals

Six- to eight-week-old Sprague-Dawley (SD) rats (Envigo, Indianapolis, IN), C57BL/6J mice (Stock # 000664), *Il10* ^-/-^ mice (Stock # 002251), *Rag* ^-/-^ mice (Stock # 008448), and *Mmp7* ^-/-^ mice (Stock # 005111, JAX, Bar Harbor, ME) were used in the experiments. Rodents were housed in the UTMB Animal Facility with a 12-hour light/dark cycle, a temperature range of 24-26°C, and a relative humidity of 40-70%. Regular chow and water were provided *ad libitum*. All animals were euthanized under deep plane of anesthesia with 4% isoflurane inhaled.

#### DSS-induced colitis models

SD rat littermates, C57BL/6J mice, and *Mmp7* ^-/-^ mouse littermates were administered 3% DSS (w/v) in drinking water for 7 days. A group of C57BL/6J mice subjected to DSS were administered anti-MMP-7 mouse monoclonal antibody (0.5 mg/kg. Cat. # MA5-43659, ThermoFisher Scientific) *via* daily *i.p.* during the entire 7-day DSS treatment and the control group received mouse IgG.

#### TNBS-induced colitis models

SD rats and C57BL/6J mice were fasted and given GoLYTELY (Braintree Laboratories, Braintree, MA) overnight to cleanse the colon. Under inhalation anesthesia with isoflurane (4% for induction and 1% for maintenance), TNBS (65 mg/kg in saline containing 40% and 10% ethanol, respectively) was administered intrarectally through a catheter (25). Animals were euthanized 7 days later.

For interventional experiment with anti-MMP-7 antibody, chronic colitis was induced in SD rats with TNBS (50 mg/kg), which was applied for 4 times, with intermittent 3-week intervals. Anti-MMP-7 rat monoclonal antibody (Cat. # 377313, Novus Biologicals, Centennial, CO) (0.1 mg/kg) was administered 3 times a week (Mondays, Wednesdays, and Fridays) *via i.p.* and the control rats received rat IgG. *In vivo* permeability assays were performed 3 weeks after the last dose of TNBS treatment, followed by euthanasia.

### CD4+ CD45Rbhi T cell transfer model of colitis

Splenic CD4+ CD45Rbhi cells (1 × 105/mouse) from selected mouse strains were i.v. transferred to Rag^−/−^ mice. Mice were sacrificed after ~15% loss of the original body weight.

### Human subjects

Ten UC patients and 10 healthy volunteers were recruited, and three physicians independently confirmed the diagnosis of UC based on clinical manifestations, serology, and endoscopy. Mucosal biopsy samples were obtained through colonoscopy. Biopsies were snap-frozen in liquid nitrogen and stored in a -80°C freezer for RNA extraction or fixed in 10% neutral buffered formalin for immunohistochemistry.

### Immunohistochemistry

Human colon biopsies and full-thickness rodent colon tissue in Swiss roll were fixed in formalin and embedded in paraffin. Five-micrometer sections were heated in a 50-55°C oven for 1 hour (26). After antigen retrieval and one-hour blocking with 10% serum, the slides with human biopsy samples were treated with anti-MMP-7 antibody (3801S, Cell Signaling, 1:50 dilution) (27) or anti-Claudin-7 antibody (bs-8482R, Bioss Antibodies, 1:50 dilution) overnight, followed by washing three times 15 min each with 1X PBS. The sections were then sequentially incubated at room temperature with biotin-labeled secondary antibody, Avidin-HRP, or DAB. For immunofluorescence detection of MMP-7 and Claudin-7 in rodent tissue, the sections treated with primary antibody were incubated for 1 hour at room temperature with ALEXA-conjugated antibody (Invitrogen) diluted 1:400 in PBS. Images were captured using a LEICA DMI 6000 B microscope. The staining intensity was scored and assigned as follows: 0 (absent), 1 (low positive signal), 2 (moderately strong positive signal), or 3 (strong positive signal).

For histologic examination, tissue sections were stained with hematoxylin and eosin (H&E). Histological score of colitis, mast cells stained with Toluidine Blue, and neutrophils and eosinophils stained with H&E, were evaluated in a blinded manner by two independent pathologists from the UTMB anatomic pathology laboratory.

### Purification of recombinant human Claudin-7

Full length human *CLDN7* cDNA was amplified by PCR and subcloned into pGEX-5X-2 vector between *BamH*I and *Sal*I restriction sites (28). PCR primers are as follows: Hu-CLDN7-F: CGT GGG ATC CAA ATG GCC AAT TCG GGC CTG CA; Hu-CLDN7-R: GAT GGT CGA CTC ACA CAT ACT CCT TGG AAG. The human Claudin-7 protein was expressed in the BL21 strain of *Escherichia coli* and purified on Glutathione-Sepharose 4B beads (MilliporeSigma). The GST tag was removed by in-column digestion with Factor Xa (MilliporeSigma).

### Claudin-7 proteolysis by MMP-7

Recombinant human Claudin-7 was incubated with active MMP-7 at a molar ratio of 5:1 at 37°C in 50 mM Tris-HCl (pH 7.5) containing 10mM CaCl_2_ and 150mM NaCl. After 15 hours, SDS sample buffer was added to stop the reaction and the samples were subjected to Western blot (WB) analysis with anti-Claudin-7 antibody.

### *In vivo* permeability assay

To evaluate colonic permeability *in vivo*, mice and rats were fasted and administered GoLYTELY overnight, followed by intrarectal injection of FITC-Dextran (MW 4,000) or Lucifer Yellow CH (MilliporeSigma) in PBS (40 mg/mL, 100 µL/mouse and 200 µL/rat) *via* a catheter advanced 3 and 8 cm, respectively, into the colon (8, 29). Two hours later, peripheral blood was taken for serum fluorescein measurement at 530 nm with excitation at 485 nm.

### Caco-2 permeability assay

Caco-2 cells were seeded into the upper chamber of a Millicell-24 cell culture insert plate at 1.5 x 10^5^/cm^2^ and allowed to grow for 14 to 21 days. The medium was changed every other day. *CLDN7*-specific siRNA was transfected using Lipofectamine RNAiMAX Transfection Reagent (Thermo Fisher). Twenty-four hours after the transfection, active MMP-7 was added to both apical and basolateral chambers and incubated for 48 hours. To assess Caco-2 monolayer permeability, Lucifer Yellow CH was added to the apical chambers and incubated at 37°C for one hour. The medium in the basolateral chambers was taken for the measurement of Lucifer Yellow flux.

### Myeloperoxidase assay

Frozen colon tissue was pulverized in liquid nitrogen, homogenized in 20 mM phosphate buffer (pH 7.4), and centrifuged at 4°C for 10 minutes. The pellets were sonicated in 50 mM phosphate buffer (pH 6.0) containing 0.5% hexadecyl trimethyl ammonium bromide and centrifuged at 4°C for 5 minutes. The supernatant (100 μL) was incubated with 16 mM tetramethyl benzidine in 50% ethanol, 0.3 mM H_2_O_2_, and 8 mM sodium phosphate buffer (pH 5.4) for 3 minutes. The absorbance was measured at 655 nm (25).

### RT-qPCR

Total RNA was extracted using the PureLink RNA Mini Kit (Thermo Fisher), followed by cDNA synthesis using M-MuLV Reverse Transcriptase (New England Biolabs, Ipswich, MA). Quantitative PCR was then performed using TaqMan Fast Advanced Master Mix or PowerUp SYBR Green Master Mix (Thermo Fisher). Glyceraldehyde 3-phosphate dehydrogenase (GADPH) or 18S rRNA served as internal controls (8, 25). Primer sequences are listed in **Table S1**.

### Western blot

WB was performed as described previously (8, 25). Primary antibodies were as follows: anti-Claudin-1 rabbit polyclonal (Cat. # A2196, 1:1000) (ABclonal, Woburn, MA), anti-Claudin-7 rabbit polyclonal (Cat. # bs-8482R, 1:1000) (Bioss Antibodies, Woburn, MA), anti-Claudin-8 rabbit polyclonal (Cat. # PIPA124415, 1:1000) (Fisher Scientific), anti-Claudin-15 rabbit polyclonal (Cat. # bs-13753R, 1:1000) (Bioss Antibodies), anti-β-actin mouse monoclonal (Cat. # MA5-15452, 1:3000) (Thermo Fisher), and anti-GADPH rabbit polyclonal (Cat. # 5174S, 1:1000) (Cell Signaling, Danvers, MA). Blots were scanned using an Odyssey Infrared Imaging System (LI-COR Biosciences, Lincoln, Nebraska). Band density was determined using LI-COR Image Studio Software.

### Statistical analysis

Data were expressed as mean ± SD. The comparisons of means among groups were analyzed by one way ANOVA, and the Dunn Multiple Comparison Test was further used to determine significant differences between groups. All statistical analyses were performed using the SPSS package (version 13.0). A value of *P <* 0.05 was considered statistically significant.

### Study approval

The Institutional Animal Care and Use Committee of the University of Texas Medical Branch at Galveston approved all procedures performed on animals (Protocol # 1512071A). The study protocol with human subjects was approved by the Ethics Committee of Binzhou Medical University Hospital (Binzhou, China).

## Results

### MMP-7 is highly expressed in the colonic mucosa of patients with UC and rodents with experimental colitis

**Figure 1:**
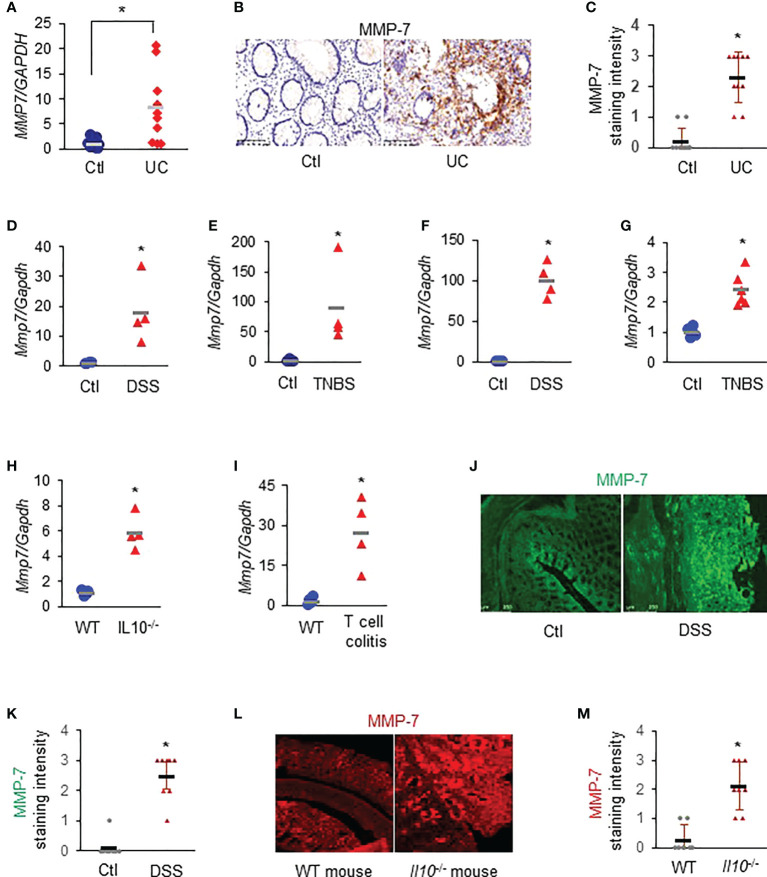
MMP-7 is highly expressed in patients with ulcerative colitis (UC) and in rodents with experimental colitis. **(A)** RT-qPCR analysis of *MMP7* mRNA levels in mucosa biopsies from control subjects and patients with UC (*n*=10). **(B)** Representative images of immunostaining for MMP-7 in human colon biopsy samples. **(C)** Quantification of MMP-7 staining intensity, ranging from absent (0) to highest (3). RT-qPCR also showed up-regulation of *Mmp7* mRNA expression in the colons of DSS rats **(D)**, TNBS rats **(E)**, DSS mice **(F)**, TNBS mice **(G)**, IL10^-/-^ mice **(H)**, and T-cell transfer colitis mice **(I)**. n=4 or 5. *p<0.01. **(J)** Immunofluorescence staining of MMP-7 in the colons of control (Ctl) and DSS rats. **(K)** MMP-7 protein staining intensity in control and DSS rats. **(L)** Representative images of MMP-7 staining of the colons from WT and *Il10* ^-/-^ mice. **(M)** Quantification of MMP-7 staining intensity in the colons of WT and *Il10* ^-/-^ mice.

RT-qPCR revealed that *MMP7* mRNA levels were significantly up-regulated in the colon biopsies of patients with UC, compared to healthy control subjects (**Figure 1A** *. p*<0.01). Immunostaining demonstrated that MMP-7 protein was also highly expressed in patients with UC, but barely detectable in control subjects (**Figures 1B, C**). To corroborate these clinical findings, we assessed *Mmp7* mRNA expression in multiple widely used murine models of colitis with different disease states using RT-qPCR. We found that the expression levels of *Mmp7* in the colon were markedly elevated in all 6 experimental colitis models examined: DSS rats (**Figure 1D**), TNBS rats (**Figure 1E**), DSS mice (**Figure 1F**), TNBS mice (**Figure 1G**), *Il10* ^-/-^ mice (**Figure 1H**), and adoptive T-cell transfer mice (**Figure 1I** *. p*<0.01). MMP-7 protein over-expression was also validated in DSS rat (**Figures 1J, K**) and IL-10 knockout mouse (**Figures 1L, M**) colon by immunostaining. Our findings pointed to MMP-7 involvement in IBD pathogenesis and warranted further mechanistic elucidation.

### TNFα, IL-1β, IL-4, IL-13, and LPS stimulate MMP-7 expression in different cells of the colon

**Figure 2:**
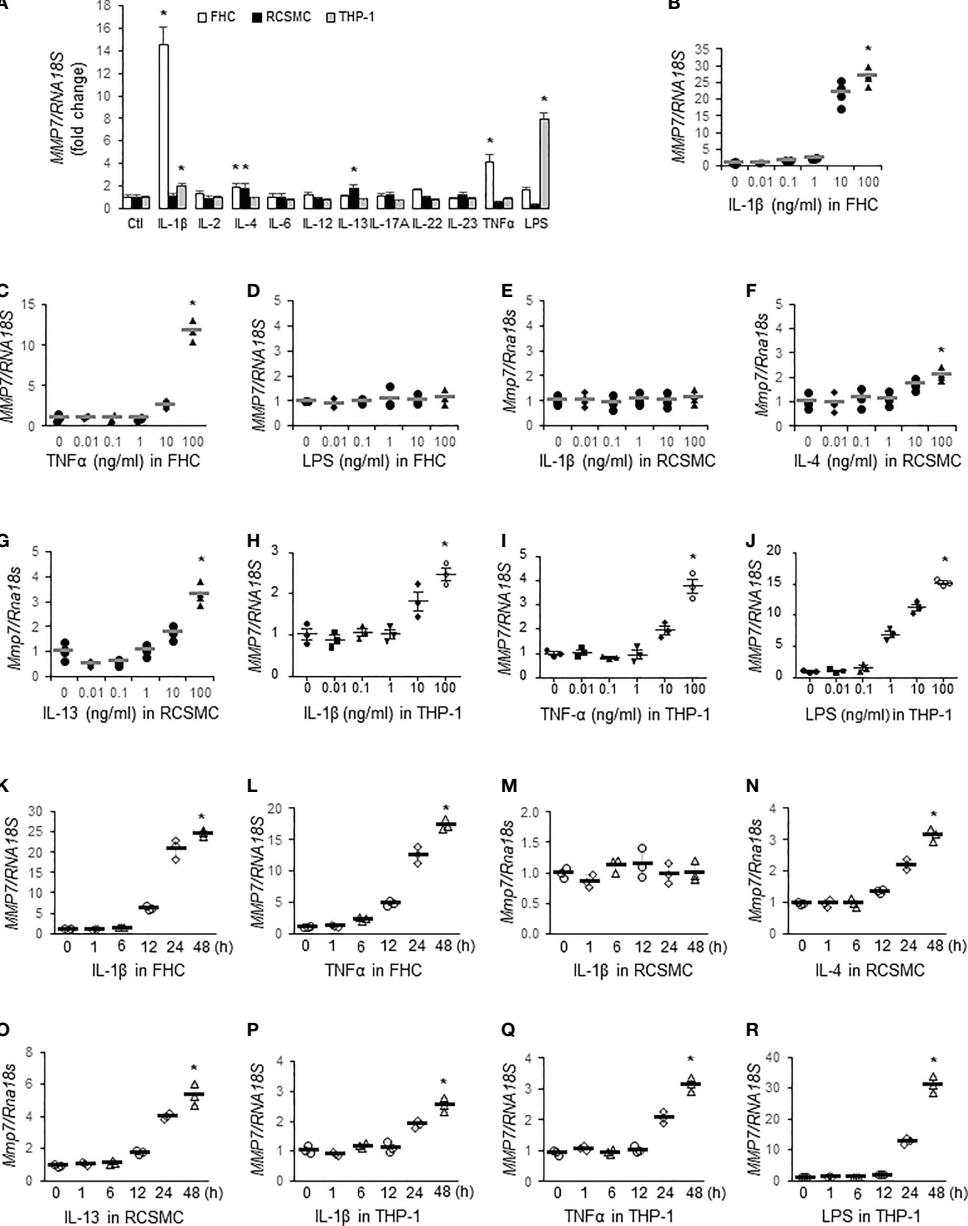
MMP-7 is up-regulated by inflammatory mediators in epithelial cells, smooth muscle cells, and macrophages of the colon. **(A)** RT-qPCR analysis of *MMP7* mRNA levels in human FHC (white bars), rat colonic smooth muscle (RCSMC, black bars), and human THP-1 (gray bars) cells treated with 10 ng/mL indicated cytokine or LPS. RT-qPCR assays also determined the *MMP7* mRNA levels in the FHC cells treated with serial doses of IL-1β **(B)**, TNFα **(C)**, or LPS **(D)**, in RCSMC treated with IL-1β **(E)**, IL-4 **(F)**, or IL-13 **(G)**, and in THP-1-derived macrophages treated with IL-1β **(H)**, TNFα **(I)**, or LPS **(J)** for 24 hours. Time course experiments were performed to evaluate temporal changes of *MMP7* mRNA expression in FHC cells treated with 10 ng/ml IL-1β **(K)** or 100 ng/ml TNFα **(L)**, in RCSMC treated with 10 ng/ml IL-1β **(M)**, 100 ng/ml IL-4 **(N)**, or 100 ng/ml IL-13 **(O)**, and in THP-1 cells treated with 10 ng/ml IL-1β **(P)**, 10 ng/ml TNFα **(Q)**, or 100 ng/ml LPS **(R)**. n=3 or 4. *p<0.01.

To identify inflammatory mediators contributing to MMP-7 over-expression in IBD, we first incubated FHC fetal human colon epithelial cells, rat colon smooth muscle cells (RCSMC), and THP-1 human macrophages with pro-inflammatory cytokines (10 ng/mL) commonly over-expressed in IBD (30), including IL-1β, IL-2, IL-4, IL-6, IL-12, IL-13, IL-17A, IL-22, IL-23, and TNFα, or LPS (10 ng/mL) and assessed *MMP7* mRNA levels by RT-qPCR. In FHC cells, IL-1β and TNFα markedly increased *MMP7* mRNA expression, whereas IL-4 and LPS marginally activated *MMP7* (**Figure 2A**, white bars. *p*<0.05). In primary culture of rat colon smooth muscle cells, both IL-4 and IL-13 significantly augmented *Mmp7* expression (**Figure 2A**, black bars). In human macrophages differentiated from THP-1 cells, IL-1β and LPS significantly up-regulated *MMP7* (**Figure 2A**, gray bars). To validate our findings, dose response experiments were performed with these 3 types of cells. RT-qPCR confirmed that IL-1β (**Figure 2B**) and TNFα (**Figure 2C**), but not LPS (**Figure 2D**), elevated *MMP7* in FHC cells in a dose-dependent manner. Neither IL-1β (**Figure 2E**) nor TNFα (data not shown) had any effect on *Mmp7* expression in RCSMCs. IL-4 (**Figure 2F**), as well as IL-13 (**Figure 2G**), concentration-dependently increased *Mmp7* in RCSMCs. In THP-1-derived macrophages, dose-dependent up-regulation of *MMP7* by IL-1β (**Figure 2H**), TNFα (**Figure 2I**), and LPS (**Figure 2J** *. p*<0.01) was observed. Time course experiments were also carried out to assess temporal changes of *MMP7* mRNA expression in all 3 types of cells. In FHC cells, 10 ng/ml IL-1β (**Figure 2K**) and 100 ng/ml TNFα (**Figure 2L**) time-dependently increased *MMP7* mRNA expression and significant increases appeared as early as 12 hours. However, 10 ng/ml IL-1β had no effect on *Mmp7* expression in RCSMCs at all 5 time points (**Figure 2M**). IL-4 (**Figure 2N**) and IL-13 (**Figure 2O**) at 100 ng/ml elevated *Mmp7* mRNA expression in RCSMCs in a time-dependent manner. In THP-1 cells, 10 ng/ml IL-1β (**Figure 2P**), 10 ng/ml TNFα (**Figure 2Q**), and 100 ng/ml LPS (**Figure 2R**) all time-dependently elevated *MMP7* mRNA levels. In both RCSMC and THP-1 cells, no significant increase of MMP7 was observed until 24 hours. These results suggested that *MMP7* was expressed not only in immune cells, but also in other major cell types of the colon wall, under inflammatory conditions such as IBD.

### MMP-7 impedes intestinal epithelial integrity by cleavage of Claudin-7

**Figure 3:**
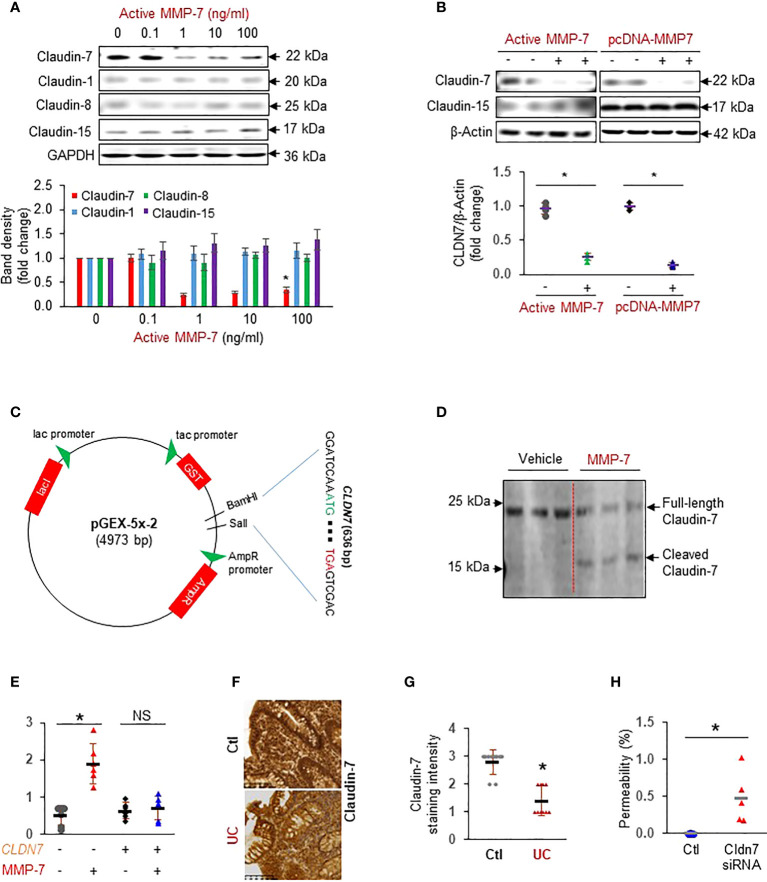
MMP-7 increases colonic epithelial permeability by degrading Claudin-7. **(A)** Representative Western blots showing that active MMP-7 dose-dependently decreased Claudin-7 in FHC cells. **(B)** Active MMP-7 and forced expression of MMP-7 suppressed Claudin-7 in CCD841 CoN cells. **(C)** Schematic presentation of pGEX-5x-2-CLDN7 construct used for purification of full-length human Claudin-7 protein. **(D)** Active MMP-7 cleaved recombinant Claudin-7 protein in enzyme assay followed by WB. **(E)** Increased Caco-2 monolayer permeability by active MMP-7 was abrogated by Claudin-7 over-expression. n=6. *p<0.01. **(F)** Representative images of immunohistochemical staining of Claudin-7 in mucosal biopsy samples from control subjects and patients with UC. **(G)** Quantification of Claudin-7 staining intensity in the human mucosal biopsies, ranging from absent (0) to highest (3). **(H)** Claudin-7 knock down by *Cldn7* siRNA increased Caco-2 permeability. n=5. *p<0.01. NS, not significant.

To test our hypothesis that MMP-7 over-expression disrupts the epithelial tight junction, we incubated FHC colonic epithelial cells with repeated doses of active MMP-7 and detected claudins commonly expressed in the colon (13). We found that MMP-7 at 1 ng/mL or higher concentrations significantly decreased Claudin-7, but not Claudin-1, Claudin-8, or Claudin-15 protein expression (**Figure 3A**). To validate that MMP-7 attenuated Claudin-7 in intestinal epithelial cells, we added 10 ng/mL recombinant human MMP-7 protein or over-expressed human *MMP7* gene in CCD841 CoN cells, another human colonic epithelial cell line. Addition or forced expression of MMP-7 markedly down-regulated Claudin-7 in CCD841 CoN cells (**Figure 3B**), confirming that MMP-7 suppresses Claudin-7 in colonic epithelial cells. To examine if MMP-7 cleaves Claudin-7, we cloned the full length human *CLDN7* gene sequence into pGEX-5X-2 vector (**Figure 3C**), purified Claudin-7 protein, incubated the protein with active MMP-7, and performed WB analysis of the mixture with anti-Claudin-7 antibody. Full-length Claudin-7 (22 kDa) was clearly cleaved by MMP-7, resulting in a ~ 16 KDa fragment, which demonstrated Claudin-7 as a novel substrate of MMP-7 (**Figure 3D**). To show that MMP-7 has a direct impact on intestinal epithelial integrity, we performed Caco-2 monolayer permeability assays in the presence or absence of 10 ng/mL active MMP-7. Compared to the vehicle control, MMP-7 significantly increased Caco-2 monolayer permeability and the increase was almost completely abrogated when Claudin-7 was stably over-expressed, strongly suggesting that MMP-7 disrupts the tight junctions by cleaving Claudin-7 (**Figure 3E**. *p*<0.01). We also evaluated Claudin-7 protein levels in colon biopsies of UC patients with high MMP-7 expression. Immunohistochemistry revealed that UC patients had decreased Claudin-7 expression, compared to the control subjects (**Figures 3F, G**. *p*<0.05), establishing an inverse correlation between MMP-7 and Claudin-7. To demonstrate that loss of Claudin-7 impairs the intestinal epithelium, we knocked down Claudin-7 in Caco-2 cells with siRNA specific to the human *CLDN7* and measured Caco-2 monolayer permeability. When compared to control siRNAs, *CLDN7* siRNAs significantly increased Caco-2 permeability (**Figure 3H**. *p*<0.01), suggesting that Claudin-7 was essential in maintaining intestinal epithelial integrity.

### MMP-7 deficiency ameliorates DSS-induced colitis

**Figure 4:**
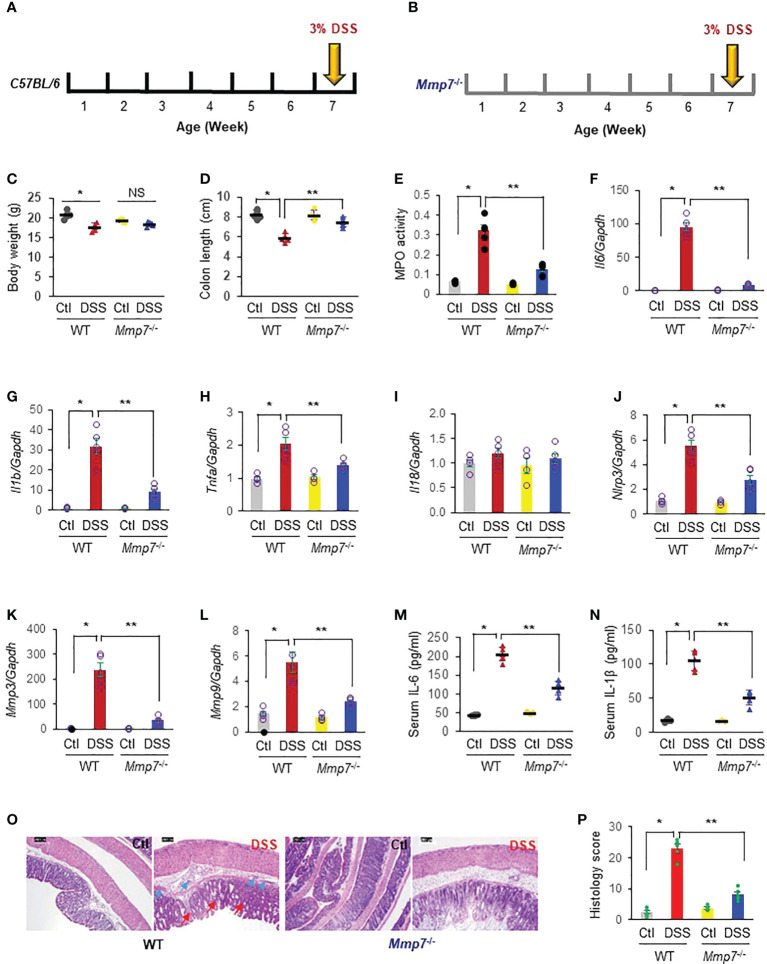
MMP-7 deficiency ameliorates DSS-induced colitis. Colitis was induced with 3% DSS in drinking water in C57BL/6 **(A)** and *Mmp7* ^-/-^ **(B)** mice for 7 days. **(C)** Body weight. **(D)** Colon length. Colon tissue was collected for detection of myeloperoxidase (MPO) activity **(E)** and mRNA levels of inflammation-related genes. Messenger RNA (mRNA) levels of *Il6* **(F)**, *Il1b* **(G)**, *Tnfa* **(H)**, *Il18* **(I)**, *Nlrp3* **(J)**, *Mmp3* **(K)**, and *Mmp9* **(L)** were quantified by RT-qPCR. Serum levels of IL-6 **(M)** and IL-1β **(N)** were assessed by ELISA. **(O)** Histologic staining **(H, E)** of the colons. Green arrow, inflammatory infiltrate. Red arrow, distorted crypt. **(P)** Histological colitis score of the colons. n=4 or 5. *p<0.05 vs wild type control. **p<0.05 vs wild type mice with DSS. NS, not significant.

We next induced colitis with 3% DSS in 6-week-old C57BL/6 wild type mice and age-matched *Mmp7* ^-/-^ mice (**Figures 4A, B**). After 7-day DSS treatment, WT mice had a significant weight loss, whereas *Mmp7* ^-/-^ only suffered slight weight loss (**Figure 4C**). DSS also caused significant colon shortening in WT mice, but not in *Mmp7* ^-/-^ (**Figure 4D**). The MPO activity was elevated >5-fold by DSS in C57BL/6 WT mouse colon, but by only 2-fold in *Mmp7* ^-/-^ mouse colon, compared to the respective controls, suggesting that MMP-7 knockout protected against colitis (**Figure 4E**). Marked increases of *Il6* (**Figure 4F**), *Il1b* (**Figure 4G**), and *Tnfa* (**Figure 4H**) mRNA were observed in WT mouse colon whereas this was less dramatic in *Mmp7* ^-/-^ mouse colon. DSS was not able to induce *Il18* mRNA expression in either WT or *Mmp7* ^-/-^ mice (**Figure 4I**). In WT mice, there was marked up-regulation by DSS of *Nlrp3* (**Figure 4J**), *Mmp3* (**Figure 4K**), and *Mmp9* (**Figure 4L**) mRNA levels, and MMP-7 knockout significantly reduced the elevation. Serum levels of secreted IL-6 (**Figure 4M**) and IL-1β (**Figure 4N**) protein were also dramatically upregulated in response to DSS treatment in WT mice; the increases were significantly ameliorated by MMP-7 depletion. H&E staining revealed that DSS-treated WT mice had significant alterations in the overall mucosal architecture (**Figure 4O**), including increased thickness, enlarged gaps, and irregular crypts, as well as markedly elevated histological colitis scores (**Figure 4P**). Cryptitis, crypt abscesses, and erosions were also observed in some areas. In contrast, DSS-treated *Mmp7* ^-/-^ mice had little alterations in the overall mucosal architecture, compared to untreated littermates, indicating mild inflammation.

### MMP-7 knockout mitigates DSS-induced changes in junction proteins

**Figure 5:**
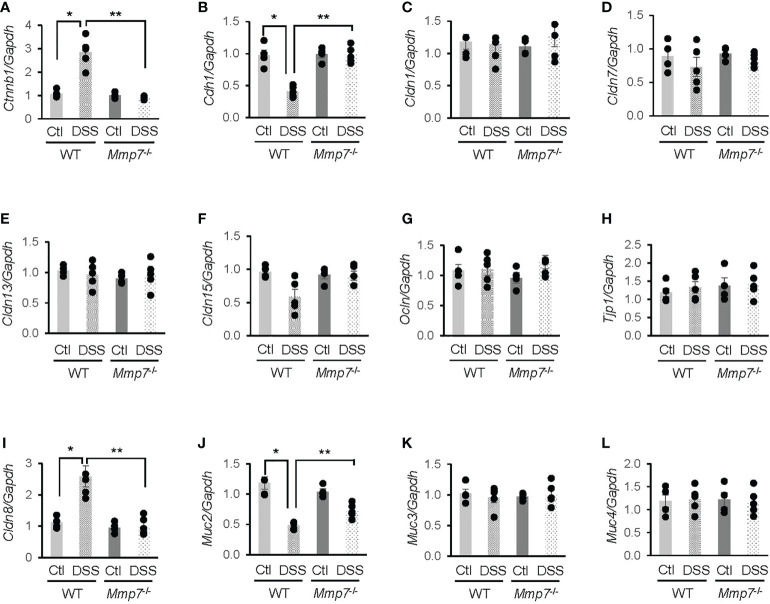
Differential mRNA expression of junction proteins in the colons of wild type and *Mmp7* ^-/-^ mice treated with 3% DSS. Colitis was induced with 3% DSS in drinking water in both C57BL/6 and *Mmp7* ^-/-^ mice for 7 days as shown in **Figure 4**. RT-qPCR was performed to determine mRNA levels of *Ctnnb1* **(A)**, *Cdh1* **(B)**, *Cldn1* **(C)**, *Cldn7* **(D)**, *Cldn13* **(E)**, *Cldn15* **(F)**, *Ocln* **(G)**, *Tjp1* **(H)**, *Cldn8* **(I)**, *Muc2* **(J)**, *Muc3* **(K)**, and *Muc4* **(L)**. n=4 or 5. *p<0.05 vs wild type control. **p<0.05 vs wild type DSS.

To further investigate whether MMP-7 mediated DSS-induced damage to the intestinal epithelium, we performed RT-qPCR analyses on mRNA levels of junction proteins in WT and *Mmp7* ^-/-^ mice treated with or without DSS. In the WT colon, β-catenin (*Ctnnb1*) mRNA expression was significantly increased (**Figure 5A**), whereas E-cadherin (*Cdh1*) gene transcription was decreased (**Figure 5B**), in response to DSS treatment; these changes were not observed in *Mmp7* ^-/-^ mice. DSS had little effect on mRNA levels of tight junction proteins Claudin-1 (*Cldn1*, **Figure 5C**), Claudin-7 (*Cldn7*, **Figure 5D**), Claudin-13 (*Cldn13*, **Figure 5E**), Claudin-15 (*Cldn15*, **Figure 5F**), Occludin (*Ocln*, **Figure 5G**), and tight junction protein 1 (*Tjp1*, **Figure 5H**) in both WT and *Mmp7* ^-/-^ mice, indicating that MMP-7 did not modulate gene transcription of most tight junction proteins. Interestingly, *Cldn8* mRNA levels in WT mice were significantly up-regulated by DSS colitis and the up-regulation was not found in *Mmp7* ^-/-^ mice (**Figure 5I**). Among mucus-related proteins, Mucin 2 (*Muc2*) mRNA levels in WT mice were significantly decreased by DSS and MMP-7 knockout had a protective effect (**Figure 5J**); no changes in *Muc3* (**Figure 5K**) or *Muc4* (**Figure 5L**) mRNA expression were found in WT or *Mmp7* ^-/-^ mice.

**Figure 6:**
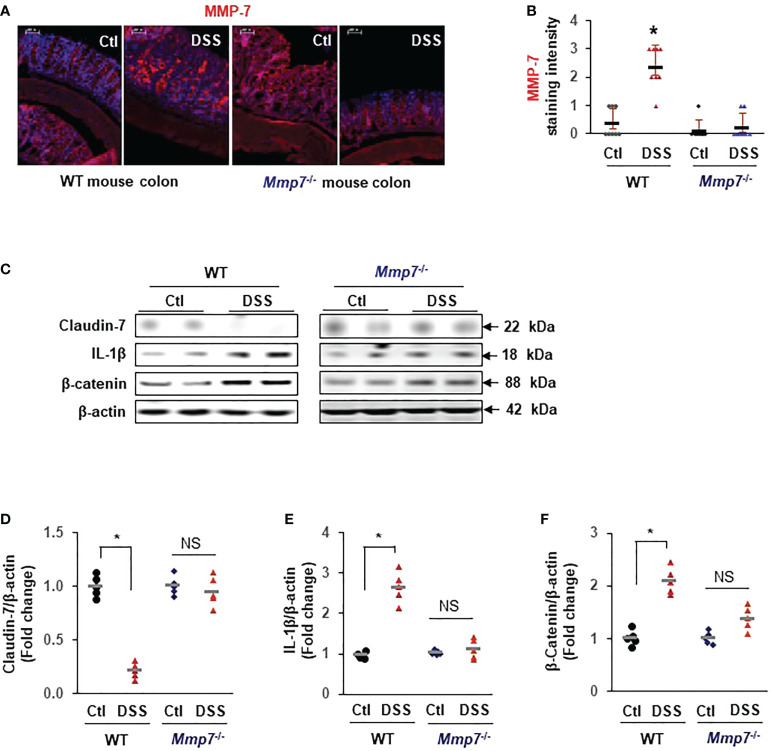
MMP-7 knockout prevents DSS-induced suppression of Claudin-7 in the colon. C57BL/6 and *Mmp7* ^-/-^ mice received 3% DSS in drinking water for 7 days as shown in **Figure 4**. Full thickness colon tissue was fixed in formalin for immunohistochemical analysis or snap-frozen in liquid nitrogen for immunoblot analysis. **(A)** Representative images of immunofluorescence staining for MMP-7 in the colons of C57BL/6 and *Mmp7* ^-/-^ mice treated with or without DSS. **(B)** Quantification of MMP-7 staining intensity. **(C)** Representative images of WB for indicated proteins in the colon. **(D–F)** Protein expression levels determined by densitometry. Arbitrary optical density units of the targeting proteins were normalized vs β-actin and presented as fold change. n=5. *p<0.01 vs WT control. NS, not significant.

To demonstrate that MMP-7 mediated Claudin-7 reduction *in vivo*, we evaluated MMP-7 protein levels in the colons of WT and *Mmp7* ^-/-^ mice treated with or without DSS. Immunofluorescence staining confirmed that MMP-7 protein was barely detectable in the colon of WT mice but was highly expressed after treatment with DSS, particularly in the distal colon (**Figures 6A, B**). No MMP-7 was detected in *Mmp7* ^-/-^ mice treated with or without DSS, as expected. Western blots further revealed that Claudin-7 protein was markedly suppressed by DSS in the colon of WT but not *Mmp7* ^-/-^ mice (**Figure 6C, D**), which is in support of our *in vitro* findings. IL-1β and β-Catenin protein levels were significantly increased by DSS only in WT mice (**Figures 6C, E, F**).

### MMP-7 blockade mitigates inflammation in rodent models of colitis

**Figure 7:**
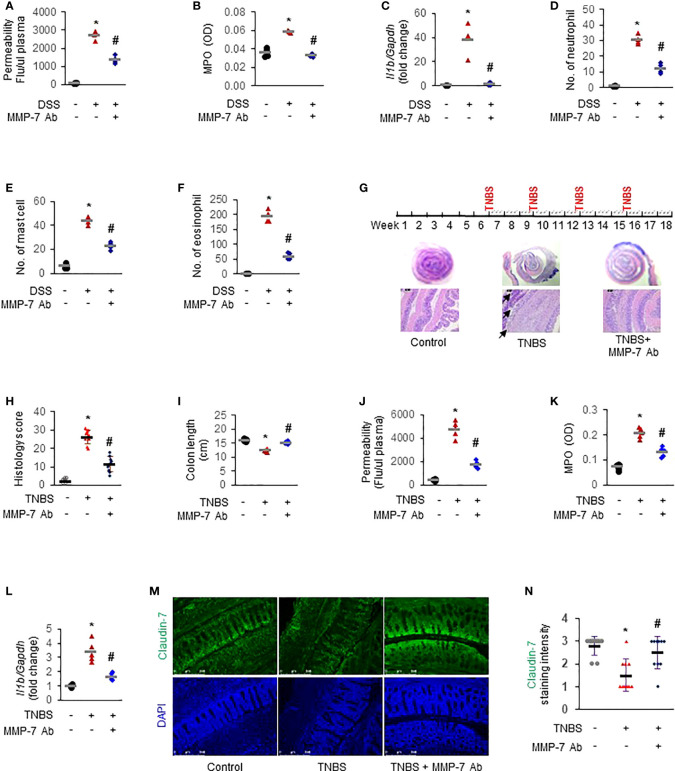
MMP-7 neutralizing antibody mitigates colonic inflammation in rodent models of colitis. For acute model mimicking ulcerative colitis, C57BL/6 mice were given 3% DSS in drinking water for 7 days. MMP-7 neutralizing antibody was administered once daily by *i.p.* injection. For chronic model mimicking Crohn’s disease, Sprague Dawley rats were treated with intrarectal injection of TNBS (50 mg/kg) 4 times, with intermittent 3-week intervals. MMP-7 neutralizing antibody was *i.p.* administered on Mondays, Wednesday, and Fridays for a total of 12 weeks. Animals in the control groups received regular drinking water (for acute model) or saline (for chronic model) as well as *i.p.* injection of respective IgG in saline. Colon tissue was fixed or snap-frozen after permeability assays. Inflammatory cells were evaluated by two independent pathologists. **(A)** *In vivo* colonic permeability of DSS mice measured with FITC-dextran at day 7. **(B)** MPO activities in the DSS mouse colon tissue. **(C)** RT-qPCR analysis of interleukin 1β (*Il1b*) mRNA levels in the DSS mouse colon tissue. **(D)** Numbers of neutrophils stained with H&E in the DSS mouse colons. **(E)** Numbers of mast cells stained with toluidine blue in the DSS mouse colons. **(F)** Numbers of eosinophils stained with H&E in the DSS mouse colons. n=4. *p<0.01 vs control (Ctl) group. #*p*<0.01 vs DSS group. **(G)** Dosing protocol (top) and H&E staining of the TNBS rat colons (bottom). Black arrow, severely damaged mucosa. **(H)** Histological colitis score of the rats. **(I)** Colon lengths of the TNBS rats. **(J)** *In vivo* colonic permeability of the TNBS rats. **(K)** MPO activities in the TNBS rat colons. **(L)** *Il1b* mRNA levels in the TNBS rat colons quantitated by RT-qPCR. **(M)** Immunofluorescence staining of Claudin-7 in colon Swiss roll sections. Nuclear counterstain was done with DAPI. **(N)** Quantification of Claudin-7 staining intensity, ranging from absent (0) to highest (3). n=5. *p<0.01 vs control (Ctl) group. ^#^ *p*<0.01 vs TNBS group.

To further establish the functional importance of MMP-7 in IBD pathogenesis and investigate the therapeutic potential of MMP-7 inhibition, we first applied MMP-7 neutralizing antibody in DSS mice with acute colitis. *In vivo* permeability assays showed that the increase of colonic permeability in response to 7-day DSS was significantly ameliorated by MMP-7 monoclonal antibody (**Figure 7A** *. p*<0.01). DSS-induced up-regulation of MPO activity (**Figure 7B**) and IL-1β mRNA expression (**Figure 7C**) was also abrogated by MMP-7 blockade. Compared to controls, there was significant, pan-colonic infiltration of neutrophils, mast cells, and eosinophils in DSS-treated mice that was abrogated by MMP-7 antibody treatment (**Figures 7D–F** *. p*<0.01). To verify the therapeutic effect of MMP-7 blockade, we administered MMP-7 antibody in rats subjected to repeat insult of TNBS, which develop transmural inflammation resembling the histopathological lesions found in human Crohn’s disease (**Figure 7G**, top) (31). H&E staining showed that TNBS induced severe hypertrophy and mucosal damage of the colon and the injury was markedly mitigated after treatment with MMP-7 monoclonal antibody (**Figures 7G, H**. *p*<0.01). TNBS-induced shortening of the colon was significantly ameliorated (**Figure 7I**, **S1A**. *p*<0.01). Increases of colonic permeability (**Figure 7J**), MPO activity (**Figure 7K**), and *Il1b* mRNA expression (**Figure 7L**. *p*<0.01) in response to TNBS were all significantly mitigated by MMP-7 antibody. Anti-MMP-7 antibody also markedly abrogated TNBS-induced neutrophil infiltration in the rat colon (**Figures S1B–D** *. p*<0.01). Of note, immunofluorescence staining revealed a significant loss of Claudin-7 in the colonic epithelium, particularly at the top of the crypts, in the rats treated with TNBS; the reduction of Claudin-7 by TNBS was rescued by anti-MMP-7 antibody (**Figures 7M, N**). These findings demonstrated that MMP-7 played a critical role in chronic gut inflammation and that MMP-7 could become a viable and highly desirable therapeutic target for IBD.

## Discussion

Patients with IBD display defects in the specialized components of mucosal barrier, including junction proteins that regulate paracellular permeability, and these defects may perpetuate chronic mucosal inflammation in IBD (32). Notably, the abnormal increase of MMP-7 in IBD was associated with the presence of mucosal erosions, architectural tissue changes, and inflammatory infiltration (33,34). However, it is not clear whether MMP-7 impairs tight junctions, a critical component of the intestinal barrier (35), by enzymatically degrading any tight junction proteins.

In this investigation, we presented compelling evidence that MMP-7 overexpression contributed to intestinal barrier impairment in IBD. We first verified MMP-7 elevation in patients with UC, as well as in multiple murine models of colitis representing various disease states of IBD. In response to inflammatory stimuli, MMP-7 was over-expressed not just in immune cells such as macrophages, but also in the epithelial cells and smooth muscle cells of the colon. Claudin-7 was identified as a novel substrate of MMP-7, and either active MMP-7 or Claudin-7 knockdown by siRNA increased Caco-2 monolayer permeability, which was further supported by studies with WT and *Mmp7* ^-/-^ mice showing that MMP-7 deficiency resulted in augmented intestinal barrier function and less severe colitis upon DSS insult. Moreover, MMP-7 antibody treatment markedly mitigated against colitis in rodents treated with DSS or TNBS, demonstrating its therapeutic potential.

By using immunohistology with surrogate markers for inflammation, neovascularization, and epithelial structures, Rath and colleagues identified endothelial cells and infiltrating leukocytes as the major cellular sources of MMP-7 and MMP-13 in UC (23). Jakubowska et al. found a strong positive correlation between MMP-7 expression in the glandular epithelium and the location of the lesion in both UC and CD patients (34). It has also been reported that MMP-7 expression levels vary along the colon (36), which is supported by our IHC data also showing differential MMP-7 levels in different areas of the colon. However, the origin of MMP-7 in the inflamed colon has not been fully characterized, partly because MMP-7 is a secreted protein with a relatively short half-life. To identify the pro-inflammatory cytokines activating MMP-7 in different cell types of the colon, we treated FHC epithelial cells established from normal fetal colonic mucosa, primary rat colon smooth muscle cells, and human THP-1-derived macrophages with LPS or 11 pro-inflammatory cytokines implicated in IBD pathogenesis (37). We identified IL-1β and TNFα as the primary activators of MMP-7 in FHC cells, whereas IL-4 and IL-13 as the major stimulators of MMP-7 in colon smooth muscle cells. In THP-1-derived macrophages, IL-1β, TNFα, and LPS augmented MMP-7 expression. Thus, IL-1β, IL-4, IL-13, TNFα, and LPS all contribute to MMP-7 accumulation in the inflamed colon, suggesting that MMP-7 blockade could be a better approach than inhibition of individual cytokines in counteracting tissue damage in IBD and other inflammatory diseases. Our data demonstrated MMP-7 activation in human epithelial cells after treatment with IL-1β or TNFα and are supported by publications showing that MMP-7 is secreted by colon and breast tumors, which are primarily epithelial cells (38, 39). However, Rath et al. detected no MMP-7 in epithelial and smooth muscle cells of human colon using immunostaining (23). It is possible the MMP-7 antibody used in their study was not able to recognize relatively lower levels of MMP-7 in epithelial and smooth muscle cells. We tested multiple MMP-7 antibodies and found that most of them working poorly in IHC. It is worth noting that smooth muscle cells, the predominant cell type in the intestine, also express MMP-7 in response to IL-4 and IL-13, which might contribute to IBD complications like perforation or fistula. Surprisingly, IL-6, which was reported to have an important role in MMP-7 expression in colonocytes (36, 40), had no effect on MMP-7 expression in our cells. Further research is required to address this discrepancy.

The increased expression and activity of MMPs are related to inflammatory state and pathogenesis of IBD (21, 41). As a matrilysin, MMP-7 degrades a variety of extracellular matrix substrates including collagens type I and IV, gelatin, elastin, fibronectin, vitronectin, laminin, entactin and tenascin, as well as non-extracellular matrix substrates, such as E-cadherin (42), Fas ligand, and Galectin-3 (22). Multiple cleavage sites have been identified for MMP-7, including AL, DL, EL, GL, KL, LL, PL, SL, PG, DY, et al. (43, 44). We reasoned that MMP-7 over-expression might contribute to the immunological imbalance of the intestinal mucosa by disrupting the tight junctions. Our results showed that the intestinal epithelial cells treated with active MMP-7 or transfected with MMP-7-expressing plasmid had significantly lower levels of Claudin-7 and increased monolayer permeability. Our enzymatic assays demonstrated that MMP-7 cleaved recombinant full-length Claudin-7 protein, identifying Claudin-7 as the first tight junction protein substrate of MMP-7. Several putative MMP-7 cleavage sites exist in Claudin-7, including G20-L21, G76-L77, A82-L83, S87-L88, G130-L131, A133-L134, P150-L151, A173-L174, and A180-L181. It is likely that active MMP-7 cleaved Claudin-7 at P150-L151 under our experimental condition because we detected a distinct band around 16 KDa using a Claudin-7 antibody with an epitope located between 21 and 100 AA of human Clauing-7 (**Figure 3D**). However, we cannot rule out that MMP-7 cleaved Claudin-7 at other cleavage sites and generated small fragments, which were not detected by this specific antibody. Decreased Claudin-7 was found in the colon biopsies of UC patients exhibiting high levels of MMP-7, which is in agreement with prior work showing reduced Claudin-7 expression in rectal biopsies of active UC (45). Furthermore, increased monolayer permeability was observed in Caco-2 cells transfected with *CLDN7* siRNAs. This is significant because Claudin-7 is a unique tight junction protein that also possesses non-tight junction functions, including maintenance of epithelial cell-matrix interactions and intestinal homeostasis (46). It has been reported that intestine specific *Cldn7* deficiency caused colonic inflammation and enhanced the paracellular permeability across the colon epithelium (17). Claudin-7 also controls intestinal crypt stem cell survival, self-renewal, and epithelial differentiation through Wnt/β-catenin signaling (20). It is mainly expressed in the colon, especially sigmoid and rectum, with little expression in the esophagus, gastric region and proximal parts of the gastrointestinal tract (47). These findings suggest that MMP-7 plays an important role in the development of IBD, at least partially, by degrading Claudin-7.

This was further supported by our findings in WT and *Mmp7* ^-/-^ mice subjected to DSS colitis. DSS induced significantly less severe inflammation in *Mmp7* ^-/-^ mice *vs.* WT mice, evidenced by less weight loss, longer colon length, lower MPO activity, and decreased mRNA levels of *Il6*, *Il1b*, *Tnfa*, *Nlrp3*, *Mmp3*, and *Mmp9* in *Mmp7* ^-/-^ mice when compared to WT mice, which is in general agreement with a previous report (48). DSS colitis also suppressed the gene transcripts corresponding to adherens junction protein E-cadherin in WT mice colon, suggesting an impairment in the epithelial barrier. In sharp contrast, the mRNA transcript levels of E-cadherin were not attenuated in the colons of *Mmp7* ^-/-^ mice, indicating a protective role of MMP-7 deletion for the cell-cell adhesion complexes. While Claudin-7 mRNA levels were not altered in both WT and *Mmp7* ^-/-^ mice, loss of Claudin-7 protein was confirmed in WT mice treated with DSS, but not in *Mmp7* ^-/-^ mice with DSS colitis (**Figure 6**), confirming posttranscriptional degradation of Claudin-7 by MMP-7.

Interestingly, β-catenin and Claudin-8 mRNA levels were up-regulated by DSS in WT mice. This could be due to the multiple physiological roles of these proteins, particularly β-catenin, which functions as a signal transducer and is actively involved in the renewal of intestinal epithelium. Higher levels of *Ctnnb1* mRNA in DSS-treated WT mice might be a result of more severe damage in the epithelium which requires rapid epithelial renewal, whereas lower levels of *Ctnnb1* in DSS-treated *Mmp7* ^-/-^ mice are due to the mild inflammation requiring less epithelial renewal. Our result seemed to be contradictory to previous findings showing that β-catenin was inhibited during colitis (49, 50). However, increased *Ctnnb1* mRNA could be a result of compensating feedback of inhibited β-catenin signaling. No changes of *Ctnnb1* and *Cldn8* mRNA were found in DSS-treated *Mmp7* ^-/-^ mice, suggesting that MMP-7 depletion helped maintain intestinal homeostasis when subjected to inflammatory insult. We also detected the mRNA levels of mucins as important components of the intestinal barrier (51, 52). Suppression of *Muc2* by DSS was mitigated in the absence of MMP-7, suggesting that MMP-7 also mediates *Muc2* reduction, similar to MMP-9 (53). Thus, both MMP-7 and MMP-9 might contribute to alteration in mucosal defenses leading to inflammation, by decreasing Muc2. Further investigation is needed to illuminate how MMPs interfere with the intestinal mucus.

MMPs are involved in cancer development, and initial drug discovery efforts primarily focused on the roles of MMPs in cancer progression. However, all of the clinical trials with more than 50 MMP inhibitors tested in various cancers failed (37). Reasons for failure may be attributed to the lack of inhibitor specificity, insufficient knowledge about the complexity of the disease biology, and relatively low levels of MMPs in tumors compared to inflamed tissue. We administered MMP-7 monoclonal antibody to rescue WT rodents subjected to DSS or TNBS insult and found that MMP-7 antibody markedly improved the intestinal barrier function, attenuated MPO activity, suppressed IL-1β expression, decreased inflammatory cell infiltration, and elevated Claudin-7 protein levels in the intestinal epithelium. The success of the MMP-7 antibody rescue experiments strongly suggests MMP-7 as a therapeutic target for IBD and perhaps other inflammatory diseases. Existing biologic therapy can alleviate inflammation in some IBD patients but sometimes fails, whereas classic immunologic agents such as infliximab have more side effects, including liver damage, respiratory problems, serious infection, and even tumorigenesis (54). MMP-7 inhibitors might offer good anti-inflammatory efficacy and limited side effects since MMP-7 is downstream of the inflammatory cascade.

In conclusion, we demonstrated that excessive MMP-7 impaired the intestinal epithelial barrier, at least partially, by cleaving Claudin-7, thereby promoting and/or exacerbating inflammation in IBD. These findings might be applicable to other inflammatory diseases, highlighting the therapeutic potential of MMP-7 inhibition as a new approach for the treatment of patients with chronic inflammation.

## Data availability statement

The original contributions presented in the study are included in the article/**Supplementary Material**. Further inquiries can be directed to the corresponding authors.

## Ethics statement

The study protocol with human subjects was approved by the Ethics Committee of Binzhou Medical University Hospital (Binzhou, China). The patients/participants provided their written informed consent to participate in this study. The animal study was reviewed and approved by The Institutional Animal Care and Use Committee of the University of Texas Medical Branch at Galveston approved all procedures performed on animals (Protocol # 1512071A).

## Author contributions

YX: Investigation, data analysis, drafting. HL: Investigation, data analysis, material support. XZ: Investigation, data analysis. YC: Material support, data interpretation, revision. RD: Data interpretation, critical revision, technical support. TS: Data interpretation, technical support, revising. DP: Data interpretation, critical revision. XL: Study concept and design, study supervision, revision. QL: Study concept and design, interpretation, obtained funding, supervision, critical revision. All authors contributed to the article and approved the submitted version.

## Funding

This work was supported in part by the National Institutes of Health [R21AI126097 and R01HL152683 to QL] and the Natural Science Foundation of Shandong Province [ZR2020MH060 to HL].

## Acknowledgments

We thank the Anatomic Pathology Laboratory at UTMB for their help with tissue embedding, cutting, H&E staining, and histological scoring.

## Conflict of interest

The authors declare that the research was conducted in the absence of any commercial or financial relationships that could be construed as a potential conflict of interest.

## Publisher’s note

All claims expressed in this article are solely those of the authors and do not necessarily represent those of their affiliated organizations, or those of the publisher, the editors and the reviewers. Any product that may be evaluated in this article, or claim that may be made by its manufacturer, is not guaranteed or endorsed by the publisher.
