# Supplementary material for: Matrix metalloproteinase 7 contributes to intestinal barrier dysfunction by degrading tight junction protein Claudin-7
Source: Front Immunol. 2022 Oct 4;13:1020902. doi: 10.3389/fimmu.2022.1020902 (PMC9581388; doi:10.3389/fimmu.2022.1020902)
Supplement: Supplementary file 1 [file DataSheet_1.pdf]

Supplemental material

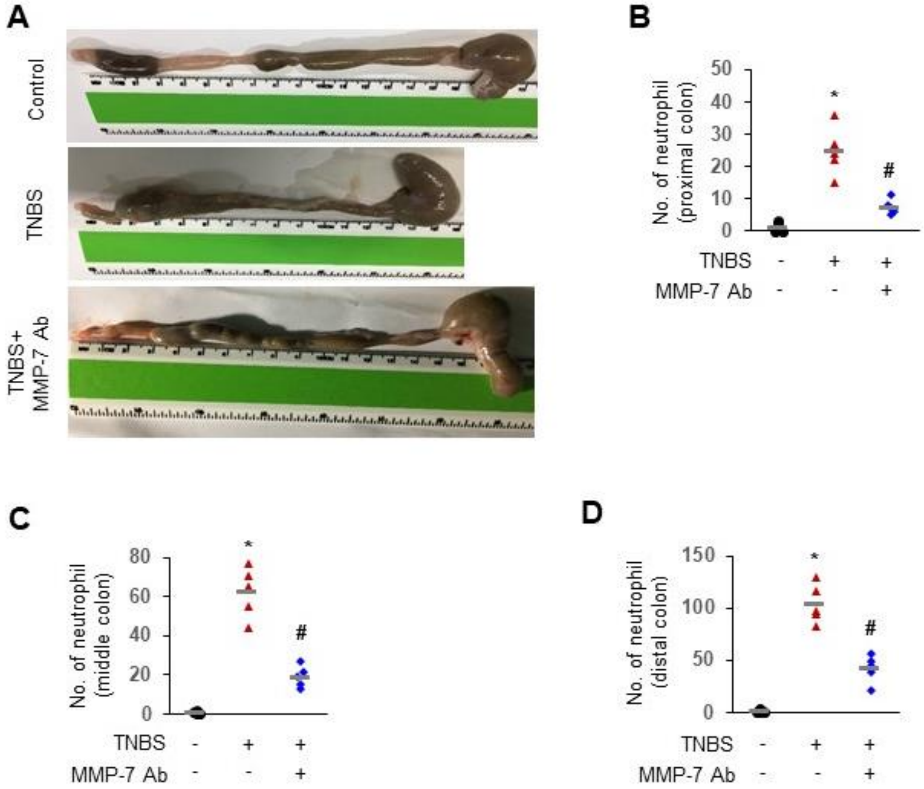

Figure S1. Anti-MMP-7 antibody ameliorates colon shortening and neutrophil infiltration in rats treated with TNBS. (**A**) Representative images of the rat colons. (**B**) Numbers of neutrophils in the proximal colons of all 3 groups of rats. (**C**). Numbers of neutrophils in the middle colons. (**D**) Numbers of neutrophils in the distal colons.

Table S1. **Primers used in SYBRGreen-based RT-qPCR.**

Gene name	Primer name	Primer sequence
Human *MMP7*	MMP7-F	5’-ACCAACTGGCCCTAAAAGGATT-3’
	MMP7-R	5’-AGAGGAATGTCCCATACCCA-3’
Human *GAPDH*	GAPDH-F	CCAGCAAGAGCACAAGAGGA
	GAPDH-R	GAGATTCAGTGTGGTGGGGG
Rat *Mmp7*	Mmp7-F	CTCTCTGGGTCTGGGTCACT
	Mmp7-R	AAGGGCGTTTGCTCATTCCA
Rat *Gapdh*	Gapdh-F	ACAGCAACAGGGTGGTGGAC
	Gapdh-R	TTTGAGGGTGCAGCGAACTT
Mouse *Mmp7*	mmp7-F	CTAGGCGGAGATGCTCACTTT
	mmp7-R	AATTCATGGGTGGCAGCAAAC
Mouse *Il1b*	Il1b-F	TTTCGTGAATGAGCAGACAGC
	Il1b-R	GTTTCTTGTGACCCTGAGCGA
Mouse *Il6*	Il6-F	ACAAAGCCAGAGTCCTTCAGAG
	Il6-R	GGGAAGCACTCACCTCTTGG
Mouse *Il18*	Il18-F	ACCCTCTCTGTGAAGGATAGTA
	Il18-R	CAAACTCCATCTTGTTGTGTCC
Mouse *Gapdh*	gapdh-F	CGTGTTCCTACCCCCAATGT
	gapdh-R	TCAGATGCCTGCTTCACCAC
Mouse *Mmp3*	mmp3-F	TTGACTCAAGGGTGGATGCT
	mmp3-R	CAACTGCGAAGATCCACTGA
Mouse *Mmp9*	mmp9-F	GAGTTCTCTGGTGTGCCCTG
	mmp9-R	TTGGAAACTCACACGCCAGA
Mouse *Muc2*	muc2-F	TCCCGACTTCAACCCAAGTG
	muc2-R	GACGGAGACAGCAGAGCAAG
Mouse *Muc3*	muc3-F	CCAGCCTTCCCTAAACCACATAA
	muc3-R	GCTAAACACGCTTCTCCTCA
Mouse *Muc4*	muc4-F	CTGCGCATCCTTCACCATCTA
	muc4-R	GATCCCGTAGAATGCCCCAAG
Mouse *Nlrp3*	nlrp3-F	CAGAAGCTGGGGTTGGTGAA
	nlrp3-R	AGTTTACAGTCCGGGTGCAG
Mouse *Ocln*	ocln-F	ACATGGCTGCTGCTGATGAA
	ocln-R	CCGTCTGTCATAATCTCCCACC
Mouse *Tjp1*	tjp1-F	TGAGCCCCCTAGTGATGTGT
	tjp1-R	GCAAAAGACCAACCGTCAGG
Mouse *Tnfa*	tnfa-F	CTTCCTCACAGAGCCAGCC
	tnfa-R	AAGACAGCTTCCCACACTGG
Mouse *Ctnnb1*	ctnnb1-F	CGAATGGATCACAAGATGGCG
	ctnnb1-R	AAGCAAAGTCAGCACCACTA
Mouse *Cdh1*	cdh1-F	GGGACAGCAACATCAGCGAA
	cdh1-R	GGATAGGTCTCACCGCCTGT
Mouse *Cldn1*	cldn1-F	TGCAAAGATGTTTTGCCACAG
	cldn1-R	ATTTGTGTAGTTTGGCAAGTATCA
Mouse *Cldn7*	cldn7-F	CTGCAACTGCTGGGCTTTTC
	cldn7-R	GGCTGTGATGATGTTGTCGC
Mouse *Cldn8*	cldn8-F	TAGTCCAGACCTCCAGGCAT
	cldn8-R	CATTCCGAGGATGGCTGTCA
Mouse *Cldn13*	cldn13-F	TGCATTGAGACCCCTAGTGC
	cldn13-R	AGTTTTCCCCTCCAGACGTG
Mouse *Cldn15*	cldn15-F	GATCACCGCCATCCTCCTG
	cldn15-R	AGATCCATGTTGCCCACGTT
Mouse Rn*18S*	rn18s-F	GTAACCCGTTGAACCCCATT
	rn18s-R	CCATCCAATCGGTAGTAGCG
